# A Low Protein Diet Increases the Hypoxic Tolerance in *Drosophila*

**DOI:** 10.1371/journal.pone.0000056

**Published:** 2006-12-20

**Authors:** Paul Vigne, Christian Frelin

**Affiliations:** 1 Institut National de la Santé et de la Recherche Médicale Nice, France; 2 Université de Nice Sophia Antipolis Nice, France; Baylor College of Medicine, United States of America

## Abstract

Dietary restriction is well known to increase the life span of a variety of organisms from yeast to mammals, but the relationships between nutrition and the hypoxic tolerance have not yet been considered. Hypoxia is a major cause of cell death in myocardial infarction and stroke. Here we forced hypoxia-related death by exposing one-day-old male *Drosophila* to chronic hypoxia (5% O_2_) and analysed their survival. Chronic hypoxia reduced the average life span from 33.6 days to 6.3 days when flies were fed on a rich diet. A demographic analysis indicated that chronic hypoxia increased the slope of the mortality trajectory and not the short-term risk of death. Dietary restriction produced by food dilution, by yeast restriction, or by amino acid restriction partially reversed the deleterious action of hypoxia. It increased the life span of hypoxic flies up to seven days, which represented about 25% of the life time of an hypoxic fly. Maximum survival of hypoxic flies required only dietary sucrose, and it was insensitive to drugs such as rapamycin and resveratrol, which increase longevity of normoxic animals. The results thus uncover a new link between protein nutrition, nutrient signalling, and resistance to hypoxic stresses.

## Introduction

Dietary restriction (DR) increases the life span in a variety of organisms such as yeasts, nematodes, fruit flies and mammals [1]–[3]. In humans, it reduces the incidence of age related chronic diseases such as diabetes, cancer and cardiovascular diseases [4]. Candidate genes that contribute to the longevity of model organisms have been identified using genetic approaches [5]–[11]. Their products are involved in insulin signalling, nutrient sensing and chromosome remodelling. It is still uncertain whether DR increases survival by the same mechanism in different organisms or after different dietary interventions [12], [13] and whether mechanisms operating in model organisms are relevant to human pathological situations. Hypoxia, for example, is a major cause of cardiac and neuronal cell death in myocardial infarction and stroke. It imposes conditions which are unique and probably not found in other pathological situations such as cancers or neurodegenerative diseases. Identifying the mechanisms which contribute to hypoxic cell death is a major objective to develop new strategies that would slow down ageing of human brains and hearts.

*Drosophila* are well suited to analyse the influence of nutrition on hypoxic tolerance (i) unlike yeast or *C. elegans*, but similar to humans, flies are obligate aerobes, (ii) large cohorts of flies can be reared to analyse demographic parameters [14], (iii) Drosophila tolerate longer exposures to hypoxic conditions than most mammals [15], [16], (iv) there is a tight conservation of hypoxic signalling pathways between flies and mammals. Hypoxia stabilises a transcription factor of the basic helix-loop-helix family (HIF-1 in mammals, Sima in Drosophila). Under normal oxygen tension, degradation of HIF-1/Sima is controlled by HIF prolyl hydroxylases, a family of 2-oxo-glutarate dependent dioxygenases that hydroxylate key proline residues in the oxygen dependent degradation domain of HIF-1/Sima. The von Hippel Lindau protein recognises hydroxylated HIF-1 proteins and targets them for proteasomal degradation. Under hypoxic conditions, activity of prolyl hydroxylases decreased, HIF-1/Sima is not anymore hydroxylated. It escapes from proteasomal degradation, migrates to the nucleus and interacts with hypoxia responsive elements in the regulatory regions of target genes [17], [18].

Here we exposed male *Drosophila* to chronic hypoxia (5% O_2_) and analysed their survival. Results indicated that survival under chronic hypoxic conditions wwas strongly dependent on dietary conditions. Maximum hypoxic tolerance only required a source of dietary carbohydrates and it was compromised by dietary proteins.

## Results and Discussion

We used male *Drosophila* for three reasons (i) their tissues are composed of postmitotic cells as are mammalian hearts and brains, (ii) their survival is independent of energy investment into egg production and (iii) their feeding behaviour seems to be independent of the quality of the food [13].

### The influence of chronic hypoxia on survival

**Figure 1:**
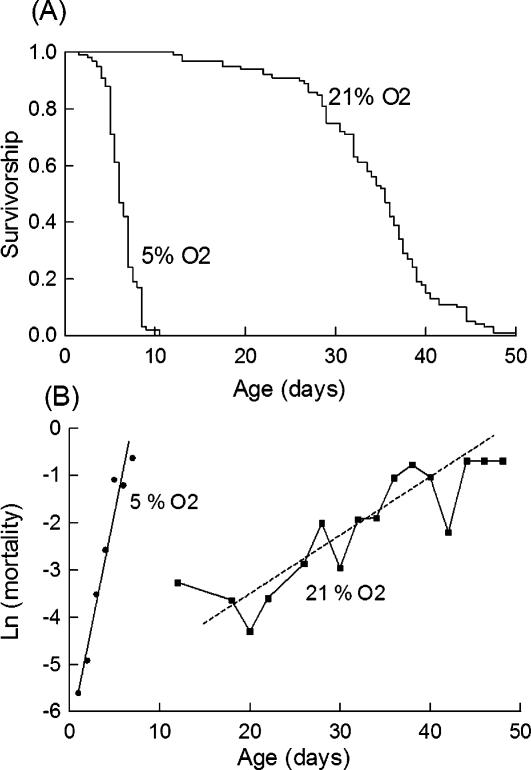
Demography of normoxic and hypoxic flies Flies fed on a 10S10Y regime were exposed to atmospheric oxygen (21% O_2_) or hypoxia (5% O_2_) as indicated. (A) Survivorship analysis. Hypoxia decreased the mean life span from 33.6±0.8 days (n = 79) to 6.3±0.1 days (n = 282). (B) Age specific mortality. Hypoxia increased five fold the slope of the representation, suggesting an accelerated ageing.

We exposed one day old flies to chronic hypoxia (5% O_2_) in the presence of a nutrient rich medium containing 10% sucrose and 10% heat inactivated yeast (abbreviated as “10S10Y”). Hypoxic flies retained a seemingly normal activity but their survival was shortened (Fig. 1A). Hypoxia decreased the average life span by >80% from 33.6 days to 6.3 days. It also decreased the median life span (from 34.5 days to 5.5 days) and the maximum life span (from 44.5 days to 8.5 days). Reduced lifespan may result from an faster age-dependent increase in mortality rate (an increase in the slope of the mortality trajectory), from an increased risk of death at all ages (a shift in the mortality trajectory), or a combination of the two. To distinguish between these possibilities we performed detailed demographic analyses. Figure 1B shows that the age specific mortalities of normoxic and hypoxic flies followed almost linear trajectories as expected from the Gompertz model. Hypoxia increased 5 fold the slope of the mortality trajectory. An usual interpretation is that the treatment increased the accumulation of irreversible damage with age [14].

Another way to accelerate ageing is to raise the temperature [14]. We therefore checked whether reducing temperature reversed the effect of hypoxia. Lowering the temperature from 25°C to 18°C decreased the activity of the flies and it increased their survival by lowering the slope of the mortality trajectory (not shown). Lowering the temperature increased the mean life span of the flies to the same extent under normoxic (2.2 fold from 33.6 days to 75.7±3.1 days, n = 89) and hypoxic conditions (2.2 fold from 6.3 days to 14.1±0.4 days, n = 194). Thus, hypoxia and elevated temperatures had independent and additive actions on the longevity of *Drosophila*.

### Dietary restriction prevented hypoxia induced death

**Figure 2:**
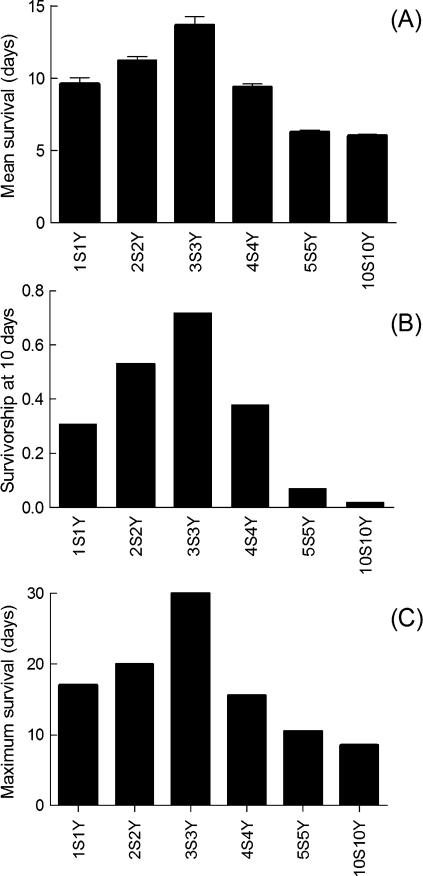
DR increased survival of hypoxic flies Influence of DR produced by food dilution on the survival of hypoxic flies . (A) Mean hypoxic life span. Means±sem are shown. (B) Short term survival measured after 10 days of chronic hypoxia. (D) Maximum survival. Sample sizes were 1S1Y (89), 2S2Y (286), 3S3Y (199), 4S4Y (280), 5S5Y (342) and 10S10Y (282).

Dietary restriction (DR) is usually applied to *Drosophila* by the simultaneous dilution of the sucrose and yeast in the nutrient medium. Figure 2 shows that hypoxic survival was strongly dependent on the composition of the diet. DR influenced the average life span (Fig 2A), the short term survival (Fig 2B), and the maximum survival (Fig 2C) in similar manners. The optimum diet was a 3S3Y diet. It increased the life span of hypoxic flies 2.2 fold (13.7±0.6 days, n = 199) as compared to a rich 10S10Y condition (6.3±0.1 days, n = 282). The net increase in life span (7.4 days) represented 22% of the average life span of normoxic flies maintained on a rich, 10S10Y diet (33.6 days).

**Figure 3:**
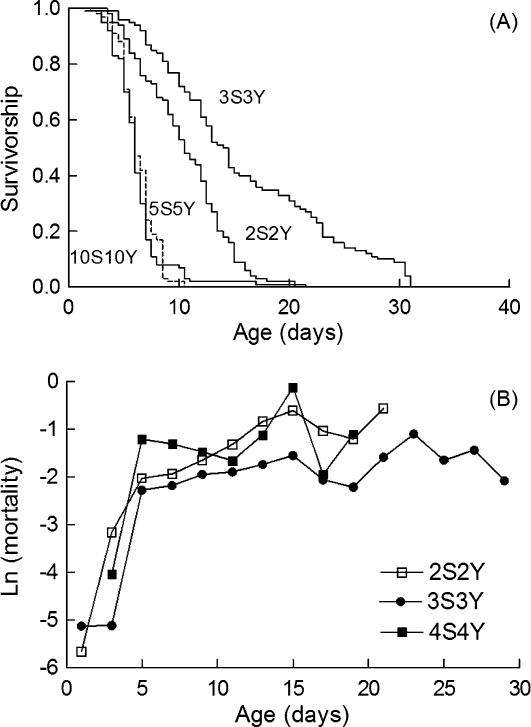
DR increased the longevity of hypoxic flies (A) Survivorship analysis of life span of *Drosophila* on different regimes as indicated. Survivorship curves corresponding to 5S5Y and 10S10Y diets were not statistically different using the log rank test. (B) Age specific mortalities for hypoxic flies exposed to 2S2Y, 3S3Y or 4S4Y diets as indicated.

Fig 3 shows selected survivorship curves and mortality trajectories of diet restricted and hypoxic flies. Mortality trajectories of flies maintained on 2S2Y, 3S3Y or 4S4Y diets deviated from linearity. During the first 4 to 6 days of hypoxia, the age specific mortality followed the same trajectory as that of hypoxic flies which were fed on a rich 10S10Y diet. Then, the mortality trajectories levelled off. One possible interpretation for these results could be that diet restricted flies adapted to hypoxia by slowing down their rate of rincrease in mortality with age. DR has a different action on normoxic flies. It reduces mortality entirely as a consequence of a lower short term risk of death [18].

### Yeast restriction reproduced the beneficial effect of DR

**Figure 4:**
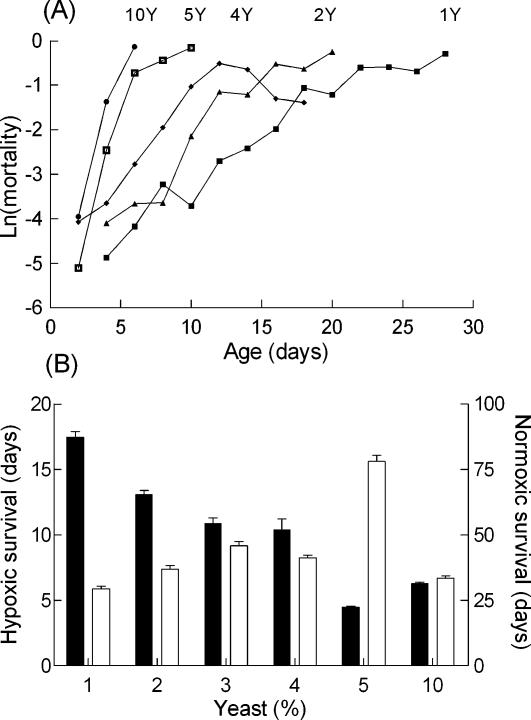
Dietary yeast promoted hypoxic death A. Mortality trajectories of hypoxic flies reared on 10% sucrose diets supplemented with different concentrations of yeast as indicated. Sample sizes were 10S1Y : 130, 10S2Y : 119, 10S4Y : 117, 10S5Y: 104, 10S10Y : 282. B. Compared actions of dietary yeast on hypoxic and normoxic flies. Flies were reared on a 10% sucrose diet supplemented with the indicated concentrations of yeast under normoxic (open symbols) or hypoxic (filled symbols) conditions and mean survivals were determined. S.e.m were smaller than the sizes of the points and are nor represented

It has previously been reported that dietary yeast plays a critical role in DR responses and that survival of normoxic flies is largely insensitive to changes in dietary sucrose [19], [20]. Similarly, restriction of dietary sucrose in the presence of 10% yeast hardly modified survival of hypoxic flies (10S10Y : 6.3±0.1 days, n = 282, 10Y : 8.3±0.1 days, n = 128). In contrasts, restriction of dietary yeast in the presence of 10% sucrose increased the average life span as much as 2.5 fold (10S10Y : 6.3±0.1 days, n = 282, 10S : 15.7±0.5 days, n = 118). The influence of yeast is further analysed in Figure 4. The panel A shows mortality trajectories of flies fed on a 10% sucrose diet in the presence of different concentrations of yeast. Yeast clearly decreased the slope of the mortality trajectory in a dose dependent manner.

**Figure 5:**
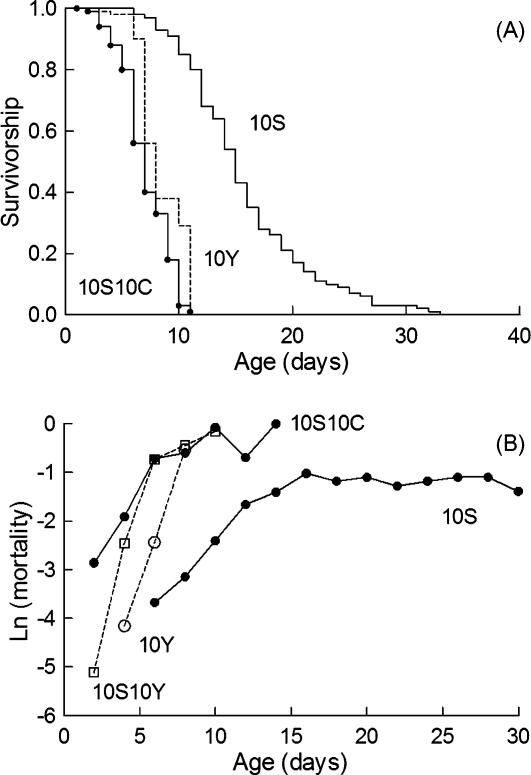
A casein hydrolysate reproduced the influence of yeast on hypoxic death Flies were reared on a pure sucrose diet (10S, n = 118), a pure yeast diet (10Y, n = 128) or on a 10% sucrose and 10% casein diet (10S10C, n = 157) as indicated. (A) survivorship analysis. (B) Age specific mortalities.

It is also of interest to note that the average life span of hypoxic flies on a yeast free and 10% sucrose diet (15.7±0.5 days, n = 118) was larger than that observed under optimum DR conditions (3S3Y : 13.7±0.6 days, n = 199). This indicated that a source of carbohydrates such as sucrose was sufficient to promote maximum hypoxic survival and that dietary yeast was toxic to hypoxic flies. It is important to stress that it is the combination of dietary proteins and hypoxia which is toxic to the flies. Dietary yeast or casein are well known to increase the life span of normoxic flies [20]–[22]. This is clearly illustrated in Figure 4B which compares the influences of dietary yeast on the survival of normoxic and hypoxic flies. As previously described by Min and Tatar [20], yeast produced a biphasic action on normoxic survival. Addition of low concentrations of yeast increased survival. Larger concentrations (>5%) decreased it. Under hypoxic conditions, yeast only decreased survival.

Finally, the observation that DR responses were reproduced by yeast restriction and not by sucrose restriction is a clear indication that the beneficial effect of DR was not related to calorie restriction, as observed previously for normoxic flies [19].

### Dietary amino acids reduce hypoxic survival

Heat inactivated yeast provides a variety of substances, including lipids and proteins. Hence to evaluate the role of dietary proteins and amino acids on hypoxic survival we used a casein hydrolysate instead of yeast. The life span of hypoxic flies on a 10% sucrose and 10% casein diet (5.9±0.2 days, n = 157) was similar to that observed on a 10S10Y diet (6.3±0.1 days, n = 282). Survivorship curves and mortality trajectories were very similar (Figure 4). Thus, a source of dietary amino acids reproduced all the inhibitory action of yeast.

Recent evidence suggest that female flies are able to change their feeding behaviour in response to changes in diet [23]. One possibility for our results could be that dietary protein inhibited feeding and induced starvation like conditions. This hypothesis was unlikely for two reasons (i) the life span of hypoxic flies fed on a protein rich diet (10S10Y : 6.3±0.1 days, n = 282) was less than that of flies exposed to complete starvation (8.6±0.3 days, n = 100), suggesting that they die before exhaustion of their reserves. (ii) Feeding of male flies was reported to be independent of diet [13] and we checked that hypoxic male flies ingested dye coloured food both in the presence and the absence of dietary proteins.

### Pharmacological evidence for a specificity of hypoxic DR responses

The mechanisms by which DR extends the life span of normoxic animals are not yet fully understood. Several candidate pathways contribute to DR responses in normoxic *Drosophila* : Sir2 [5], the insulin like signalling [6], [7] and TOR, the target of rapamycin [8]. Insulin and TOR signalling are closely linked. For instance amino acid starvation activates the TOR pathway in the larval fat body and triggers a starvation signal that modulates insulin signalling in peripheral tissues [24]. Participation of TOR to the hypoxic DR response was unlikely for two reasons (i) TOR signalling is inhibited in hypoxic flies [25], (ii) rapamycin did not promote the survival of hypoxic flies fed on a 10S10Y or a 3S3Y diet (data not shown). Further pharmacological evidence also rule out participation of sir2. Resveratrol, an activator of sir2 which extends the life span of normoxic flies [26], did not increase survival of hypoxic flies fed on a 10S10Y or a 1S1Y diet (data not shown).

### Conclusion

Our results uncover a new and unsuspected link between protein nutrition and hypoxic tolerance. Chronic hypoxia decreased the life span of male *Drosophila* and this effect can be partially reversed by restriction of dietary amino acids. Recent evidence suggests that DR improves risk factors profiles for protection against cardiovascular diseases in humans [4] and has both cardioprotective and neuroprotective actions in rodent models of ischemic diseases [27], [28]. The identification of dietary amino acids and nutrient signalling as major factors that determines hypoxic survival in the *Drosophila* model suggest novel possibilities to develop DR mimics and to reduce hypoxic cell death and its consequences in humans.

## Materials and Methods

Larvae of the w*^1118^* strain were reared on a standard diet (8.2% cornmeal, 6.2% sucrose, 1.7% yeast and 1% agar supplemented with 3.75 g/l methyl 4-hydroxybenzoate). Newly emerging adult males were collected over a 24 h period, divided into batches of 10 flies per vial and exposed to different diets. The nutrient media consisted of sucrose, heat inactivated yeast powder, 2% agar and 3.75 g/l methyl 4-hydroxybenzoate. A “10S10Y” nutrient medium means a 10% Sucrose and 10% Yeast nutrient medium. In some experiments, a casein hydrolysate was used as a source of aminoacids. Vials were sealed with rubber septa (SubA Seal, ID 22 mm, Sigma, St Louis, Mo). They were flashed with 20 volumes of a premixed 5% O_2_/95% N_2_ atmosphere and using two 18G needles. The flies were maintained at 25°C under a 12 h/12 h light/dark cycle and scored for survival twice a day. Dead flies were diagnosed by their lack of a sit-up response.

In experiments using pharmacological tools, 250 µl solutions of 100 µM resveratrol in phosphate buffered saline or of 50 µM rapamycin in diluted ethanol were layered on the top of the nutrient media and allowed to adsorb overnight. We checked that the vehicle did not modify the life span of hypoxic flies.

Mean survival±sem are indicated. Maximum survival was calculated by computing the median life span of the final surviving 10%. Data were analysed using the GraphPad prism 4 software.
